# The clinical efficacy and safety of traditional Chinese medicine in the treatment of recurrent aphthous stomatitis: A protocol of systematic review and meta-analysis

**DOI:** 10.1097/MD.0000000000022588

**Published:** 2020-10-02

**Authors:** Jiaping Lu, Naizheng Zhang, Wenhao Qian

**Affiliations:** Department of Stomatology, Shanghai Xuhui District Dental Center, Shanghai, China.

**Keywords:** protocol, recurrent aphthous stomatitis, systematic review, traditional Chinese medicine

## Abstract

**Background:**

The objective of this meta-analysis was to summarize and identify the available evidence from studies to estimate the clinical value of traditional Chinese medicine (TCM) in the treatment of recurrent aphthous stomatitis (RAS) and provides clinicians with evidence on which to base their clinical decision making.

**Methods:**

This review will include all studies comparing clinical efficacy of TCM in the treatment of RAS. The search strategy will be performed in 9 databases. We will not establish any limitations to language and publication status, published from inception to the August 2020. Two reviewers will screen, select studies, extract data, and assess quality independently. Outcome is clinical efficacy, pain relief, duration of wound healing, effect on wound healing, rate of recurrence, adverse events, and safety. The methodological quality including the risk of bias of the included studies will be evaluated. We will carry out statistical analysis using RevMan 5.3 software.

**Results:**

This study will summarize current evidence to assess the efficacy and safety of TCM in the treatment of RAS.

**Conclusion:**

The findings of this study will provide helpful evidence for the clinician, and will promote further studies, as well as studying the value of TCM.

**Registration number:**

INPLASY202080126 (DOI number: 10.37766/inplasy2020.8.0126).

## 1 Introduction

Recurrent aphthous stomatitis (RAS), also known as recurrent aphthous ulceration and canker sores, is a frequent oral mucosal disease that exhibits different geographical incidence, the incidence of RAS is 5% to 25%. RAS is known to be very painful, and it may even have a negative effect on the quality of life of the affected individual, impairing eating, swallowing, and speaking. Clinically, RAS manifests as one of three forms: minor, major, and herpetiform. The minor form constitutes 85% of all RAS, and is characterized by recurrent ulcers of <1 cm in diameter each, that heals spontaneously within 7 to 10 days without leaving scar. In the major form, the ulcers are larger, exceeding 1 cm in size; last longer, up to 1 month; and usually result in scarring. The herpetiform variant presents as clusters of many 1 to 2 mm ulcers that heal in 10 to 14 days. Treatment of RAS is quite challenging and so far there is no specific therapy.[^1^,^2^,^3^,^4^]

For centuries, Chinese medicine has been used as a complementary and alternative medicine for western medicine in China. As a public disease, RAS has a high incidence in China. In addition, Chinese clinicians have extensive experience in treating RAS, conducting a large number of clinical trials.^[5]^ Although, TCM have been used clinically in the treatment of RA for many years, the efficacy and safety still need evidence-based medical research and clinical evidence-based literature of TCM for RAS is not sufficient. Consequently, the objective of this meta-analysis was to summarize and identify the available evidence from these studies to estimate the clinical value of TCM. And provides clinicians with evidence on which to base their clinical decision making.

## 2 Methods

### 2.1 Study registry

The protocol was registered on the International Platform of Registered Systematic Review and Meta-analysis Protocols (INPLASY202080126). The preferred reporting items for systematic review and meta-analysis protocols (PRISMA) will serve as guidelines for reporting present review protocol and subsequent formal paper.^[6]^

### 2.2 Eligibility criteria for including studies

#### 2.2.1 Types of studies

We will include all studies comparing the TCM in the treatment of RAS, including observational study and RCT. Any other types of studies, such as animal studies, case reports, case series, and review will all be excluded.

#### 2.2.2 Types of interventions

##### 2.2.2.1 Experimental group

All patients in the experimental group received TCM alone or in combination with western medicine or in for their treatment in this study (including oral Chinese medicine or external application of Chinese medicine).

##### 2.2.2.2 Control group

The participants in the control group could receive any other treatments in this study.

#### 2.2.3 Types of patients

Patients suffered from RAS will be included without sex, age, course, ethnicity, disease duration, or disease severity restrictions.

#### 2.2.4 Types of outcome measurements

**Primary outcomes.** The criterion efficacy will be divided into four categories: complete remission (CR), partial remission (PR), stable (SD), and invalid (PD). The total effective rate was (CR+PR)/(CR+PR+SD+PD) × 100%.

**Secondary outcomes.**

1. pain relief (immediate and during healing);
2. duration of wound healing;
3. effect on wound healing;
4. rate of recurrence;
5. adverse events;
6. safety

### 2.3 Literature sources and search

We will perform literature searches using the following electronic bibliographic databases from their inception onwards to the August 2020: MEDLINE, Springer, Web of Science, PubMed, EMBASE, the Cochrane Central Register of Controlled Trials, Evidence Based Medicine Reviews, VIP, and CNKI. We will not establish any limitations to language and publication status. The following electronic databases were searched from their inception dates through August 2020. The search terms were integrated as follows: “∗recurrent∗ AND ∗aphthous stomatitis∗ AND (∗Traditional Chinese Medicine∗ OR ∗Traditional Chinese Medicine Formula∗ OR ∗Chinese Herb Formula∗ OR ∗Chinese herbal drug∗).”

### 2.4 Study selection

**Figure 1:**
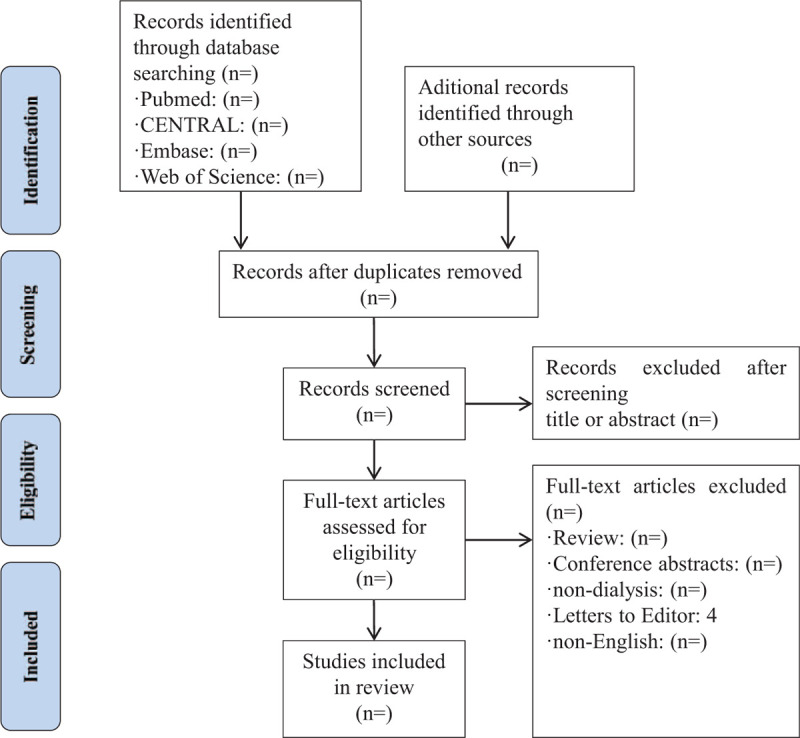
Study flow.

All duplicated studies will be imported into Endnote X7 software and excluded before the screening. Two authors will independently scan all the records from title and abstract and all irrelevant literatures will be removed. Then, full manuscripts of all remaining studies will be further identified to check if they meet all inclusion criteria. We will note all excluded citations with specific reasons. If there are any different opinions between 2 authors, we will invite another author for consultation and final decision will be made after discussion. The detail of the study selection will be presented in a PRISMA flow diagram (Fig. 1).

#### 2.4.1 Data extraction

Two authors will independently extract the following associated information from each included trial: first author, time of publication, sample size, randomization methods, blinding, concealment, allocation, details of intervention and controls, duration of follow-up, outcome measurement tools, and any other relevant information. A third senior author will help to reconcile any divergences between 2 authors.

#### 2.4.2 Missing data dealing with

If we identify any unclear or missing data, we will contact original authors to obtain them. If we cannot get reply, we will only analyze available data and will discuss its potential affect as limitation.

#### 2.4.3 Quality assessment

The Cochrane risk of bias tool, which is recommended by the Cochrane Reviewer's Handbook 5.0.2, will be used to evaluate the quality of the included studies. Two independent reviewers will evaluate the quality of selected articles from the following 5 aspects: selection bias (random sequence generation or allocation concealment), performance bias and detection bias (blinding), attrition bias (incomplete outcome data), reporting bias (selective outcome reporting), and other biases. If necessary, we will contact the corresponding author to clarify issues. The result of the consistency evaluation will be presented with Kappa statistics, Kappa value <0.75 will be considered the consistency has reached. Any disagreements will be resolved through discussion or consultation.

#### 2.4.4 Subgroup analysis

Based on available data, we will perform the following subgroup analyses: different types of treatment.

#### 2.4.5 Sensitivity analysis

We will consider running sensitivity analysis to identify the robustness and stability of merged results by excluding studies with high risk of bias.

#### 2.4.6 Reporting bias

If necessary, we will examine the reporting bias using funnel plot and Egger regression test when >10 trials are included.

### 2.5 Data synthesis

We will undertake RevMan 5.3 software to analyze data and to perform meta-analysis if it is necessary. We will calculate all continuous data using mean difference or standardized mean difference and 95% confidence intervals. As for dichotomous data, we will exert it using risk ratio and 95% CI. The heterogeneity as determined by the Cochran statistics was <0.10 of the *χ* ^2^ test. If the *I* ^2^ value was >50%, we marked it as a considerable level of heterogeneity; otherwise, we considered it to be a good homogeneity. We also assessed clinical heterogeneity. Statistically and clinically homogeneous studies were pooled using a fixed-effects model; otherwise, a random-effects model was used when the heterogeneity was significant. Additionally, subgroup analysis will be operated to explore any possible reasons for the high heterogeneity. Whenever it is possible, we will conduct meta-analysis if at least 3 eligible criteria are fulfilled. Otherwise, meta-analysis will not be carried out if only 1 or 2 studies meet the inclusion criteria. Under such situation, the findings will be presented in a narrative summary. We will perform narrative synthesis if running meta-analysis is inappropriate due to the high heterogeneity. All narrative descriptions will be carried out based on the Guidance on the Conduct of Narrative Synthesis in Systematic Reviews.

## 3 Discussion

RAS is a very common oral disease affecting a large segment of the population. Currently, the etiopathogenesis of RAS is not fully understood, but there are some suggested predisposing factors such as a family history for aphthous lesions or systemic disorders like deficiencies (e.g., Vit B12, D) with or without underlying gastrointestinal disorders, endocrine imbalance or allergies. Mouth ulcers very similar to RAS can appear in some systemic disorders, but these conditions should be distinguished from RAS. Known local predisposing factors for RAS are trauma, food hypersensitivity, dental procedures, stress, and non-smoking.[^7^,^8^,^9^]

The aim of RAS therapy is immediate pain alleviation, and ideally, therapy would decrease the frequency or even stop the onset of acute phases. Treatment modalities are topical or systemic. Topical interventions include mouth rinses, pastes, gels, sprays, injections, locally dissolving tablets, and laser treatment. The pharmacological agents proposed for topical treatment include anti-inflammatory drugs, antiseptics, antibiotics, anaesthetics, and corticosteroids. But, a commonly accepted treatment modality for RAS is still missing.[^10^,^11^,^12^,^13^] So, the objective of this study was to summarize and identify the available evidence from studies to estimate the clinical value of TCM in the treatment of RAS.

The strength of this systematic review and meta-analysis will include: search a comprehensive range of databases, including Chinese and English databases, more rigorous and detailed concerning quality assessment and data extraction. In addition, the findings obtained in the present study will provide helpful evidence in clinical practice. Furthermore, it will also help to promote further studies and clarify the direction for the future research.

In contrast, this study has several potential limitations. There may be a language bias, although there is not language limitation in this study. Moreover, there may be a large heterogeneity, which may bias the results.

## Author contributions

JPL and NZZ are co-first authors of this manuscript, contributing equally to the design and drafting the manuscript. All authors participated in the design of the study and performed it. WHQ is corresponding author of this manuscript. All authors read and approved the final manuscript.

**Conceptualization:** Jiaping Lu.

**Formal analysis:** Jiaping Lu.

**Funding acquisition:** Naizheng Zhang, Wenhao Qian.

**Investigation:** Naizheng Zhang, Wenhao Qian.

**Methodology:** Wenhao Qian.
